# Human-Automation Interaction Design for Adaptive Cruise Control Systems of Ground Vehicles

**DOI:** 10.3390/s150613916

**Published:** 2015-06-12

**Authors:** Hwisoo Eom, Sang Hun Lee

**Affiliations:** Intelligent HMI/CAD Lab, Graduate School of Automotive Engineering, Kookmin University, 77 Jeongneung-ro, Seongbuk-gu, Seoul 136-702, Korea; E-Mail: djagnltn@hanmail.net

**Keywords:** adaptive cruise control, formal verification, intelligent vehicle, mode confusion, user interface

## Abstract

A majority of recently developed advanced vehicles have been equipped with various automated driver assistance systems, such as adaptive cruise control (ACC) and lane keeping assistance systems. ACC systems have several operational modes, and drivers can be unaware of the mode in which they are operating. Because mode confusion is a significant human error factor that contributes to traffic accidents, it is necessary to develop user interfaces for ACC systems that can reduce mode confusion. To meet this requirement, this paper presents a new human-automation interaction design methodology in which the compatibility of the machine and interface models is determined using the proposed criteria, and if the models are incompatible, one or both of the models is/are modified to make them compatible. To investigate the effectiveness of our methodology, we designed two new interfaces by separately modifying the machine model and the interface model and then performed driver-in-the-loop experiments. The results showed that modifying the machine model provides a more compact, acceptable, effective, and safe interface than modifying the interface model.

## 1. Introduction

Highly automated systems, such as airplanes, can operate in various modes that can be changed directly by the pilots or automatically by the system. However, human-automation interfaces may not provide sufficient feedback on the state of automation [1,2], and human operators may not fully understand how the automation process works (*i.e.*, they may not have an accurate mental model) [3]. Both of these issues can lead to mode confusion, wherein the human operator is unable to keep track of the state or mode of the automated device. This is dangerous because mode confusion can result in surprising automated function changes, resulting in the operator’s awareness being disrupted by the automated device behaving in an unexpected manner [4,5]. Many accidents have been caused by mode confusion and unexpected automated functions when automated systems are in operation. Butler *et al.* [6] analyzed 184 aviation accidents and showed that the most common cause of flight accidents was mode confusion in pilots using automated systems, followed by incorrect operations.

Recently, intelligent vehicles have been equipped with adaptive cruise control (ACC) systems to improve driver and passenger safety. ACC systems enhance conventional cruise control systems and are capable of controlling the speed of a vehicle as set by the driver. This allows a host vehicle to follow a forward vehicle at an appropriate distance by controlling the engine and/or power train and potentially the brake. Recently, machine-learning algorithms have been introduced for personalized ACC systems in which the gap value is set automatically and customized to the driving styles and preferences of the driver as well as the driving conditions [7,8]. As with other highly automated systems, ACC systems operate in multiple modes that may cause the driver to experience mode confusion or automation surprise. Therefore, it is necessary to consider methods of reducing the possibility of mode confusion during the design of driver interfaces for ACC systems [9].

There has been substantial research on mode confusion and the resulting automation surprise in highly automated systems, particularly in aircrafts [1,2,3,4,5,6]. Several recent studies have focused on mode confusion in ACC systems [10,11,12,13,14,15,16,17]. Formal methods have been used widely to evaluate the properties of human-automation interfaces and determine whether there is potential for mode confusion via automated theorem proving or model checking. In particular, Degani and Heymann [10,11] proposed a formal methodology for the generation and analysis of user interfaces. They identified inaccurate mental models as one of the major causes of mode confusion and presented a step-by-step procedure for generating information content for an interface that is both correct and succinct. However, mode confusion during human interactions with automated systems can be attributed to not only incorrect metal models (*i.e.*, the driver misunderstanding the behavior of the automated function) but also complexity (*i.e.*, unnecessarily complex automation) and opacity (*i.e.*, poor display of the automation state). Therefore, it is necessary to develop a holistic design approach for the user interfaces of automated systems by considering various sources of mode confusion.

To meet this requirement, we developed a new interface design methodology for automated systems and verified its effectiveness through user interface design and driver-in-the-loop experiments with ACC systems. In this paper, we first propose criteria for the correctness of the interface model of an automated system, which represents the operational modes, events, and transitions between modes in the user interface. We then suggest a simple approach using a state and mode transition table for a combined machine and interface model of the system to determine whether an interface model satisfies these criteria. We also propose an interface-centric design approach in which the interface model is designed first and the machine model is adapted to it. Finally, through driver-in-the-loop experiments, we verify that this approach provides a more acceptable and effective user interface than the machine-centric approach, in which the machine model is implemented first and the user interface is adapted to it.

This paper is organized as follows. Section 1 presents the background and objectives of this study. Section 2 summarizes a survey of previous research related to mode confusion and driver interfaces in ACC systems. Section 3 presents a methodology for the design and verification of a user interface based on a formal analysis method. Section 4 describes a traditional machine model, a standard interface model, a conventional graphical user interface, and the proposed combination of the machine and interface models. Section 5 presents three candidates for the ACC user interface that were developed from the interface proposed in Section 4 by applying the design methodology presented in Section 3. Section 6 describes the driver-in-the-loop experiments conducted in a simulated environment. Section 7 summarizes the experimental results in detail. Section 8 and Section 9 present the discussions and conclusions drawn from the results of this study.

## 2. Literature Survey

Formal methods comprise a set of well-defined mathematical languages and techniques for the specification, modeling, and verification of systems [18]. Specifications are formulated to describe desirable system properties using rigorous, unambiguous notations. Thus, systems can be modeled using mathematically based languages that support well-established theoretical formalisms, such as finite state automata, directed graphs, Büchi automata, Petri nets, and μ-calculus. The verification process mathematically proves whether the model satisfies the specifications using two main methods, automated theorem proving and model checking [5]. Formal verification has been used successfully in a number of applications whose performance must be guaranteed, particularly computer hardware. Formal verification has also been used to evaluate the properties of human-automation interfaces. In the majority of these analyses, the human-automation interface behavior is modeled formally, and the properties related to the behavior of the interfaces are checked against this model. A particular subset of human-automation interface analyses is concerned with discovering whether there is potential for mode confusion, and several approaches have been proposed to address this specific problem.

Degani and Heymann [10,11] proposed a formal approach and methodology for the analysis and generation of user interfaces, with a special emphasis on human-automation interaction. In this method, the correspondence between the behavior of the machine and the abstracted information provided to the user can be formally described and analyzed by considering the machine, the user’s tasks, the user interface, and the interface model of the machine. They also presented a step-by-step procedure for generating information content for the interface that is both correct and succinct, and they explained and illustrated the procedure using two examples. They identified inaccurate mental models as one of the major causes of mode confusion. However, they did not discuss the mode confusion caused by vague or inappropriate displays of the system status on the user interface. This type of problem is related to human cognition, and it cannot be detected using formal methods; thus, the possibility of mode confusion must be verified experimentally.

More specifically, mode confusion in ACC systems has recently been investigated by several researchers. Horiguchi *et al.* [12,13] showed that if different modes exhibit similar responses, users have difficulty distinguishing among them. They proposed a new method that uses mode vectors to estimate the possibility of mode confusion. Each mode is encoded into vector form from the perspective of its input-output relations before the distances between the vectors are calculated and used as indices to estimate the similarity of the modes. To demonstrate the validity of this method for predicting possible mode confusion, the researchers calculated the similarities between ACC modes using this method and compared the results to experimental results obtained using a driving simulator. To avoid similarities between mode vectors, which result in mode confusion, the method requires that some extra outputs be added to modes that are represented by the same vector.

Furukawa *et al.* [14] conducted an experimental study of mode awareness using a dual-mode ACC system with high- and low-speed modes. They identified information that could be effective in supporting mode awareness in complex situations if some direct information concerning the system state is concealed. Two experiments were conducted. The results of the first experiment showed that a clear visual display of the system state is highly effective in reducing mode confusion. The results of the second experiment showed that, contrary to common belief, overlapping the ranges of the high- and low-speed modes improves not only the ease of transition between modes but also the drivers’ mode awareness.

Heymann and Degani [15] described a hierarchy of automated driving aids and their functionalities, beginning with standard cruise control (CC) and followed by ACC with the option of full-speed range functionality to allow stopping and starting before the addition of automatic lane centering and the possible future incorporation of navigation functions. They provided a formal description of each system and illustrated the likely effects of the incremental growth in functionality and other advances on the corresponding user interface. They stated that these formal models are advantageous in terms of precision and rigor in characterizing how automated systems are operated by drivers (by enabling formal testing, simulation, and verification). They presented formal models of operation and suggested display concepts to facilitate efficient interaction. However, they did not implement formal models, and their displays were not tested in driver-in-the-loop experiments for the purpose of verification.

Lee *et al.* [9,16,17] studied possible mode confusion in a simulated environment in which vehicles were equipped with an adaptive cruise control system. They developed a new driver interface for ACC systems based on a formal method and conducted a set of driver-in-the-loop experiments to observe possible instances of mode confusion and redesign the user interface to reduce their occurrence. The experimental results showed that the clarity and transparency of the user interface was as important as the correctness and compactness of the mental model in reducing mode confusion. However, their approach resulted in a complicated user interface because they separated the modes of the user model to eliminate incompatible mode transitions that caused mode confusion. In addition, they did not present any well-established criteria to verify the correctness of the user interface. Thus, to overcome these limitations, it is necessary to develop a new design methodology for compact and correct user interfaces of highly automated systems, particularly ACC systems, and to experimentally verify its effectiveness.

## 3. Proposed Methodology for the Design and Verification of User Interfaces

**Figure 1:**
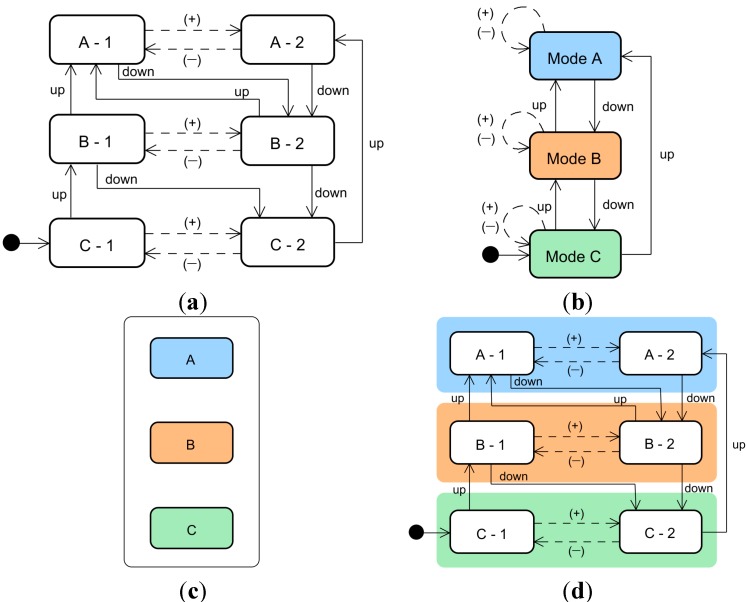
Machine and interface models and a user interface for an example machine: (**a**) machine model; (**b**) initial interface model; (**c**) initial user interface; and (**d**) combined machine and interface model.

Human-machine interactions can be formally described and analyzed by considering the following four elements: the machine, the user’s tasks, the user interface, and the user’s model of the machine. In general, a machine is modeled as a state transition system [12]. The state transition model of a machine is commonly referred to as a machine state model; in this paper, we use the term “machine model”. A state represents a certain internal configuration of the machine, and the transitions represent discrete state changes that occur in response to triggering events. Some of these transitions occur only if triggered by the user, whereas others are triggered automatically. For instance, Figure 1a illustrates a sample machine model with six states and their transitions. In this paper, user-triggered transitions are depicted as solid lines, and automatically triggered transitions are depicted by dashed lines. The transition lines are directed and labeled according to the triggering event that causes the machine to transition from one state to another. Most machines do not show all of the internal states or events to users. Thus, the internal states and their transitions are clustered and abstracted before they are displayed in the user interface. We refer to this cluster of states as a mode. From the user’s viewpoint, a machine is recognized via a user interface as a mode transition system that comprises modes, events, and transitions among modes. This mode transition model of the interface is commonly referred to as an interface mode model; in this paper, we use the term “interface model”. The interface model is equivalent to the user model defined in Reference [12]. The user interface is the obtained result of implementing this interface model. Figure 1b and c show an interface model and its corresponding user interface, respectively.

To prevent users from experiencing mode confusion and automation surprise during human-machine interactions, the interface model must be correct, and the user interface must provide suitable feedback about the state of the system. We propose the following two criteria for use in analyzing and evaluating the correctness of interface models. These criteria can be expressed mathematically and applied using formal analysis methods [11].

- The response of the machine to user-triggered events must be deterministic; that is, when starting in the same mode, identical user events should produce identical transitions between system modes.
- Mode changes that are not present in the interface model must not be triggered by users.

To determine whether an interface model satisfies these criteria, we propose a simple approach that uses a state and mode transition table for a combined machine and interface model that contains all states and modes, all events triggered by the user or system, and their resulting transitions. Two states must be distinguished from each other if they do not satisfy all of the following conditions: (a) They belong to the same mode; (b) Each user-triggered event that is available and active in one state is also available and active in the other state; (c) Starting from each of the two states, the same triggering event results in post-transition states that also satisfy conditions (a) and (b). The state pairs that satisfy these conditions are referred to as compatible [12]. Thus, the verification of an interface model design begins with building a state and mode transition table and proceeds with determining whether these criteria are satisfied.

If incompatible states are found in a mode, any of the following three approaches can be employed to resolve the incompatibilities: (1) Modify the interface model by partitioning the mode such that incompatible states are in separate modes; (2) Modify the states and/or their transitions in the machine model; (3) Simultaneously modify the modes of the interface model and the states of the machine model. The second one is a user-centric design approach in which users’ interest has priority over that of engineers, whereas the first one is an engineer-centric design approach in which engineers’ interest has priority over that of users. The third one is a hybrid approach, which is usually adopted when the second approach cannot be implemented due to limited possibilities for modification of the machine model.

**Figure 2:**
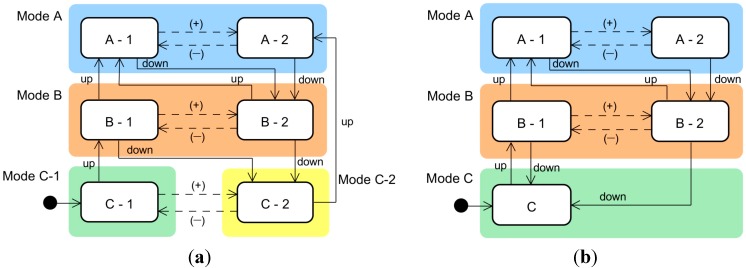
Two different approaches to modifying a user interface so that it satisfies the mode confusion criteria: (**a**) modifying the interface model; and (**b**) modifying the machine model.

For example, if we apply the above criteria to the user interface shown in Figure 1, the state and mode transitions occur as shown in Table 1. Thus, states C-1 and C-2 are incompatible because they do not satisfy condition (c). That is, as illustrated in Figure 1d, for the user operation “up”, states C-1 and C-2 are transitioned to states B-1 and A-2, respectively, which belong to different modes. Because the transitioned state pairs resulting from the same triggering event do not belong to the same mode, states C-1 and C-2 are not compatible. To resolve this incompatibility, we can employ one of the three approaches mentioned above. For example, if we adopt the first approach (modifying the interface model), mode C is partitioned to ensure that each state belongs to an independent mode. The modified interface model is shown in Figure 2a, and its state and mode transition table is given in Table 2a. Alternatively, if we employ the second approach (modifying the machine model), state C-2 is eliminated, and the transitions to and from it are modified according to the interface model. The modified interface model is shown in Figure 2b, and its state and mode transitions are given in Table 2b. In the following sections, our proposed methodology is applied to the design and verification of the ACC user interface.

**Table 1:** User-triggered state and mode transitions in the example machine.

Mode	State	User’s Operation		Compatible
		Up	Down	
A	A-1		B-2	Yes
	A-2		B-2	Yes
B	B-1	A-1	C-2	Yes
	B-2	A-1	C-2	Yes
C	C-1	B-1		No
	C-2	A-2		No

**Table 2:** State and mode transitions of the modified interface model for the example machine: (**a**) modifying the interface model; and (**b**) modifying the machine model.

**(a)**					
**Mode**	**State**	**User’s Operation**			**Compatible**
		**Up**		**Down**	
A	A-1			B-2	Yes
	A-2			B-2	Yes
B	B-1	A-1		C-2	Yes
	B-2	A-1		C-2	Yes
C1	C-1	B-1			Yes
C2	C-2	A-2			Yes
**(b)**					
**Mode**	**State**		**User’s Operation**		**Compatible**
			**Up**	**Down**	
A	A-1			B-2	Yes
	A-2			B-2	Yes
B	B-1		A-1	C-2	Yes
	B-2		A-1	C-2	Yes
C1	C-1		B-1		Yes
C2	C-2		A-2		Yes

## 4. Machine and Interface Models of ACC Systems

### 4.1. Traditional Machine Model of ACC Systems

**Figure 3:**
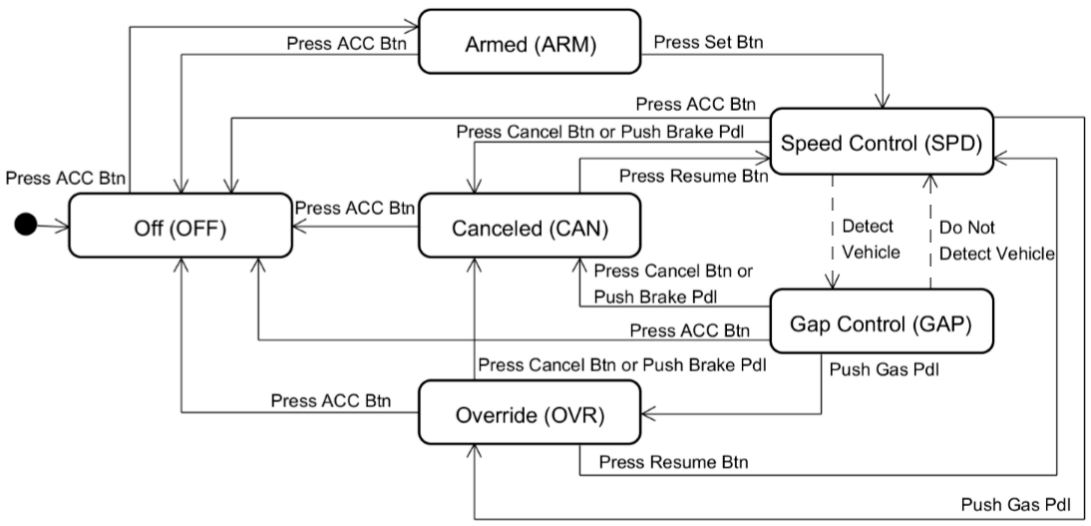
Traditional machine model of an ACC system composed of six states.

As illustrated in Figure 3, the machine model of the ACC systems implemented in most current vehicles comprises six states: off, armed, canceled, override, speed control, and gap control [17]. The armed state is a standby state that is turned on by pressing the ACC button and waits for the activation of the speed control state, which is performed by pressing the set button. The canceled state is similar to the armed state except that its previous state is the gap control or speed control state and the driver presses the resume button to return from the canceled state to the speed control state. The armed and canceled states are frequently confused by the driver because different buttons should be pressed to enter the speed control state from each of the two states. The override state is a temporary state in which the driver takes control of the vehicle from the ACC by pressing the gas pedal when the system is in the speed or gap control state. The speed control state is an active state in which no forward vehicles are present and the ACC system maintains the vehicle speed at the set speed, as is typical with conventional cruise control systems. The gap control state is also an active state in which the time gap, or headway, between the ACC vehicle and the target vehicle is being controlled.

The user triggers include pressing and releasing the brake and gas pedals; pressing various buttons to activate, cancel, and resume the operations of the ACC system; and increasing and decreasing the set speed and set gap distance. When the ignition key is in the off position, no power is supplied to any of the systems. When the key is cycled to the on position, the ACC system initializes in the off state. Before the active ACC can be engaged, the driver must first enter the armed state by pressing the ACC on button. The driver enters the speed control state by pressing the set or resume button. If a prior set speed is present in the memory, the system uses this prior value as the target speed when the resume button is pressed; otherwise, the current speed of the vehicle when the set button is pressed will become the target speed. When entering active ACC control, the vehicle speed is controlled to maintain either a set speed or a time gap with respect to a forward vehicle, whichever yields a lower speed [19].

### 4.2. Traditional Interface Models of ACC Systems

**Figure 4:**
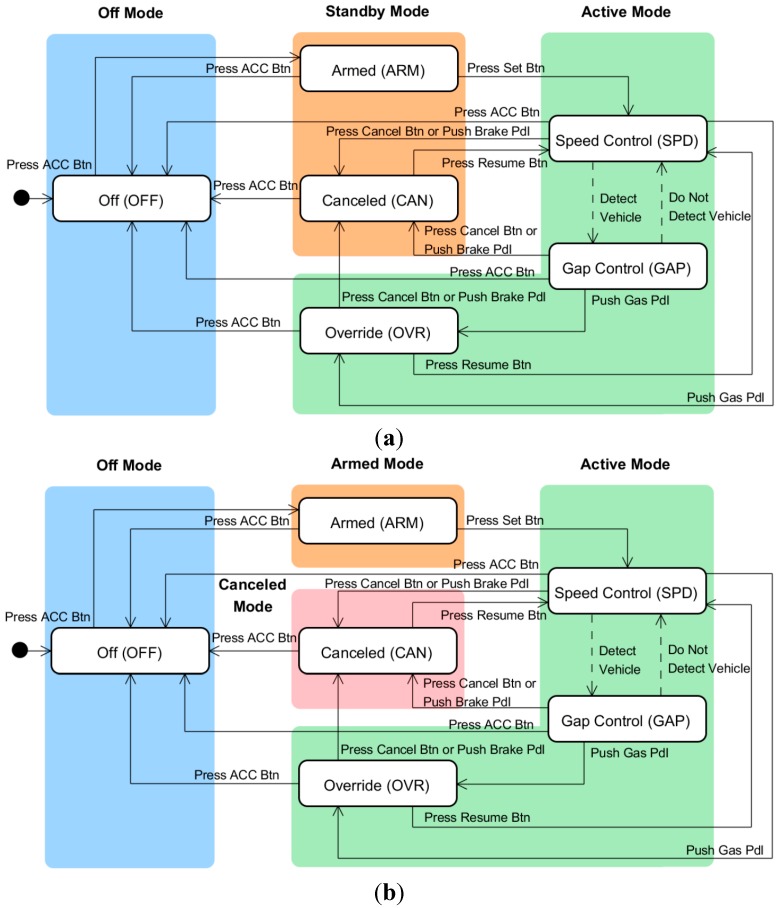
Typical traditional ACC interface models: (**a**) three-mode interface model [17] (by permission of the Korean Society of Mechanical Engineers); and (**b**) four-mode interface model.

Different designers and companies group states into different modes, with each mode being a cluster of states sharing the same functionality and display. The interface models with three and four modes, shown in Figure 4, are typical traditional models that are widely adopted by major automotive manufacturers, including Hyundai and Toyota [20,21]. The three-mode model includes off, standby, and active modes, whereas the four-mode model includes off, armed, canceled, and active modes. In both models, the gap control, speed control, and override states belong to the active mode. The armed and canceled states belong to the standby mode in the three-mode model and to the armed and canceled modes, respectively, in the four-mode model. The state and mode transition tables of the two interface models, which are shown in Table 3, indicate that incompatible mode transitions exist in the standby and active modes. The three-mode model is an old ACC interface style and has been proven to cause a high rate of mode confusion due to incompatible mode transitions [17]. Therefore, we adopted the four-mode interface model as a baseline for comparison with the performance of newer models.

**Table 3:** State and mode transitions of the traditional ACC interface models.

Interface Model	Mode	State	Driver’s Operation							Compatible
			Press ACC Button	Press Set Button	Press Resume Button	Press Cancel Button	Push Brake Pedal	Push Gas Pedal	Release Gas Pedal	
Three-mode Interface	Off	OFF	ARM							Yes
	Standby	ARM	OFF	SPD						No
		CAN	OFF	SPD	SPD					No
	Active	OVR	OFF			CAN			SPD	No
		SPD	OFF			CAN	CAN	OVR		Yes
		GAP	OFF			CAN	CAN	OVR		Yes
Four-mode Interface	Off	OFF	ARM							Yes
	Armed	ARM	OFF	SPD						Yes
	Canceled	CAN	OFF	SPD	SPD					Yes
	Active	OVR	OFF			CAN			SPD	No
		SPD	OFF			CAN	CAN	OVR		Yes
		GAP	OFF			CAN	CAN	OVR		Yes

## 5. Development of New Interface Models for ACC Systems

To solve the incompatibility problem in the traditional interface models, we apply the proposed method to the ACC machine model shown in Figure 3. The design process consists of the following three steps.

Step 1 Design the machine and interface models: The states in the machine model and the modes in the interface model are designed based on common sense, and the states are then grouped into the appropriate modes.
Step 2 Test the compatibility of the two models: Any incompatible mode transitions that may cause mode confusion in drivers are detected.
Step 3 Redesign the machine and interface models: If any incompatible mode transitions exist, the interface and/or machine models are modified to eliminate the incompatible modes using the methods described in Section 3.

### 5.1. Design of Machine and Interface Models

**Figure 5:**
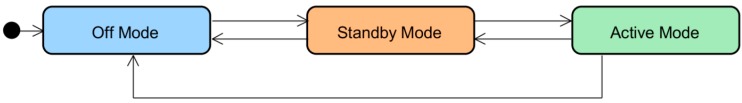
Three-Mode interface model of an ACC system.

We began with the ACC interface model presented in the test specifications of ISO 15622:2010 [19]. This model has three modes (active, standby, and off), and their transitions via user-triggered events are shown in Figure 5.

**Figure 6:**
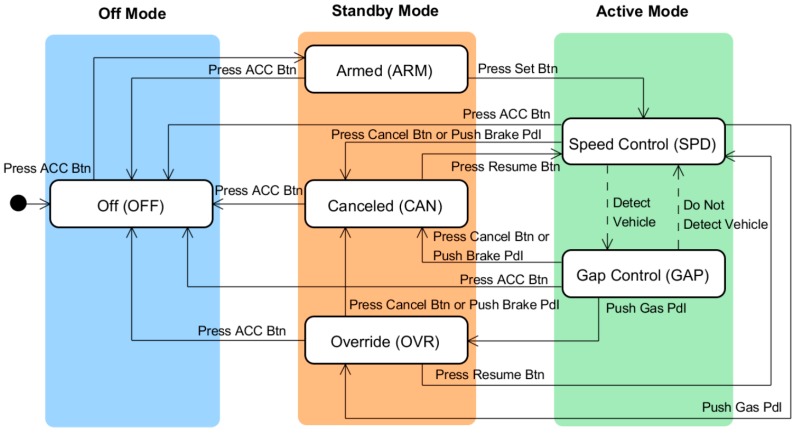
Grouping of the machine model states in the three-mode interface model.

We grouped the states of the machine model into the modes of the interface model so that the meaning of each state was consistent with that of the corresponding mode. The gap control and speed control states were assigned to the active mode, which corresponds to when the ACC system controls the vehicle speed or clearance. The armed, canceled, and override states were assigned to the standby mode, which corresponds to when the system waits for activation caused by the driver pressing a button to start or resume system operation. The off state was assigned to the off mode, which corresponds to when the system is turned off. Figure 6 shows the results after the interface model reconfiguration.

### 5.2. Compatibility Test of Machine and Interface Models

We determined whether there were any incompatible mode transitions in the interface model proposed in the previous step using the state and mode transition table shown in Table 4. There were no problems with the transitions from states in the off and active modes. There is only one state in the off mode, so inconsistencies in this mode were impossible. The gap control and speed control states, which comprise the active mode, made transitions into the same modes via user-triggered events. However, incompatible mode transitions occurred in the standby mode. The override, canceled, and armed states in the standby mode did not satisfy conditions (b) and (c), which must be satisfied by states in the same mode, as described in Section 3, resulting in incompatible mode transitions that lead to mode confusion. More specifically, when the resume button is pressed in the standby mode, if the system was in the canceled state it transitions to the speed control state in the active mode, whereas if it was in the armed state, it remains in the same state; that is, the same user trigger in the same mode causes transitions into different modes. Because these two states in the same mode have the same display, the driver cannot readily distinguish between them. Therefore, the driver may experience mode confusion or automation surprise because of the unexpected incompatible modes produced by the same user operation. For example, the driver may believe the system is in the active mode after he presses the resume button in the armed state, although the system actually remains in the armed state. When a vehicle in the left lane abruptly moves in front of the driver’s vehicle, he expects his vehicle will slow down automatically to maintain the defined gap distance. However, because his vehicle remains in standby mode, the system does not reduce its speed, possibly resulting in a crash.

**Table 4:** User-triggered transitions between states and modes in the proposed interface model.

Interface Model	Mode	State	Driver’s Operation							Compatible
			Press ACC Button	Press Set Button	Press Resume Button	Press Cancel Button	Push Brake Pedal	Push Gas Pedal	Release Gas Pedal	
Initial Interface	Off	OFF	ARM							Yes
	Standby	ARM	OFF	SPD						No
		CAN	OFF	SPD	SPD					No
		OVR	OFF			CAN			SPD	No
	Active	SPD	OFF			CAN	CAN	OVR		Yes
		GAP	OFF			CAN	CAN	OVR		Yes

### 5.3. Redesign of Machine and Interface Models

To resolve the incompatibilities in the interface model shown in Figure 6, as described in Section 3, we can employ any of the following three approaches: (1) a machine-centric approach of modifying the modes of the interface model with no change to the machine model; (2) an interface-centric approach of modifying the states and/or their transitions in the machine model with no change to the interface model; or (3) a hybrid approach of simultaneously modifying the modes of the interface model and the states of the machine model. In this study, we adopted the first and second approaches to design two user interfaces with five and three modes as potential solutions to the incompatibility state problem of the initial model.

**Figure 7:**
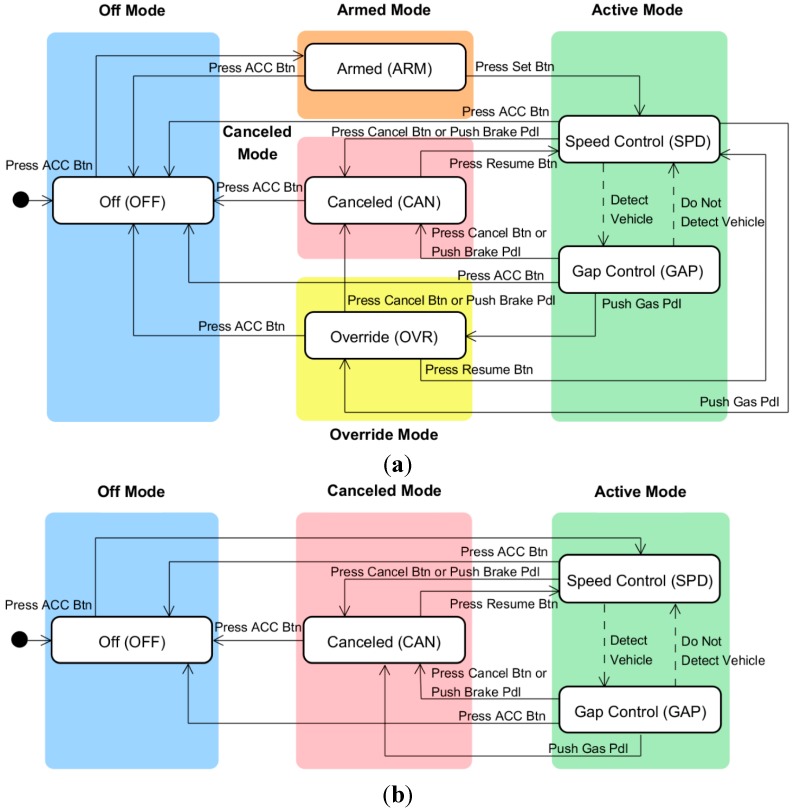
Design of new interface models for the ACC system: (**a**) five-mode interface model; and (**b**) three-mode interface model.

First, following the machine-centric approach, we divided the standby mode into separate modes for each incompatible state. Thus, as shown in Figure 7a, the standby mode was partitioned into the override, canceled, and armed modes, which include the override, canceled, and armed states, respectively. The state and mode transition table of the new user interface, shown in Table 5, demonstrates that the incompatible mode transitions are eliminated by this mode division. In our previous study [17], this proposed model was evaluated and compared with the traditional three-mode interface model shown in Figure 4a. The five-mode interface showed a lower rate of mode confusion than the conventional three-mode interface. However, the five-mode interface has such numerous overly detailed modes that it may be difficult for drivers to learn how to use it and become familiar with it. This complication arises because we accepted the traditional machine model and then designed a compatible interface model without modifying the machine model. This strategy restricted the freedom of the interface design and prevented us from developing a user-centric system. Norman [22] defined user-centric design as “a philosophy based on the needs and interests of the user, with an emphasis on making products usable and understandable”. He explained that products are usable and understandable when the user can figure out what to do and tell what is going on. Therefore, we expected that the system designed by the interface-centric approach would be more user-friendly and increase the usability which plays an important role in the acceptability of the system from user [23].

**Table 5:** User-triggered transitions between states and modes in the new interface models.

Interface Model	Mode	State	Driver’s Operation							Compatible
			Press ACC Button	Press Set Button *	Press Resume Button	Press Cancel Button	Push Brake Pedal	Push Gas Pedal	Release Gas Pedal	
Five-mode Interface	Off	OFF	ARM							Yes
	Armed	ARM	OFF	SPD						Yes
	Canceled	CAN	OFF	SPD	SPD					Yes
	Override	OVR	OFF			CAN			SPD	Yes
	Active	SPD	OFF			CAN	CAN	OVR		Yes
		GAP	OFF			CAN	CAN	OVR		Yes
Three-mode Interface	Off	OFF	SPD							Yes
	Canceled	CAN	OFF		SPD					Yes
	Active	SPD	OFF			CAN	CAN	CAN		Yes
		GAP	OFF			CAN	CAN	CAN		Yes

* not available in the three-mode interface.

To investigate the effect of the user-centric design, we designed a three-mode interface model by following the interface-centric approach. In the machine model, shown in Figure 3, if the driver turns on the ACC system by pressing the ACC button, the system enters the armed state, which is a ready state that waits for specific activations by the driver. The driver then presses the set button to switch the system state to speed control. Once the system enters the speed control state, it never returns to the armed state. Therefore, we removed the armed state and let the off state transition to the speed control state directly when the ACC button is pressed. In addition, many drivers are confused by the canceled and override states. When the brake pedal is pressed, the ACC system transitions from the gap control and speed control states to the canceled state, where it remains after the brake pedal is released. Conversely, when the gas pedal is pressed, the system transitions to the override state and returns to the previous (gap control or speed control) state when the gas pedal is released. The different responses to the pedal operations can confuse the driver. To solve this problem, we removed the override mode and made the speed control and gap control states both transition to the canceled state when either the gas or brake pedal was pressed. The resulting machine model is illustrated in Figure 7b. Next, we determined whether there were any incompatible state transitions in the state and mode transition table for the modified machine model, which is shown in Table 5, and found that there were no incompatible transitions in the model. For all states in the same mode, the same user-triggered events result in transitions to states in the same post-transition mode.

## 6. Driver-in-the-Loop Experiments

### 6.1. Participants

In this study, 40 participants, consisting of 10 females and 30 males aged between 20 and 65 years (mean = 36.53 years, SD = 13.67), were selected from the student population of Kookmin University and a private company. Information was obtained from the participants using a basic questionnaire. Every participant had a valid driver’s license and one or more years of driving experience but little experience with CC or ACC systems. All participants had normal or corrected-to-normal vision.

### 6.2. Procedure

Before the experiments began, the participants filled out consent forms and a basic questionnaire. They were then informed about the goal of the study and the experimental methods. The experimenter explained the ACC system in detail, including its operational modes and states. Next, each participant obtained hands-on experience with the ACC system in the driving simulator. It took 20 min for participants to complete the process that preceded the experiment, *i.e.*, completing the consent form and practice driving.

The experiments with the three-, four-, and five-mode models were conducted in different orders for different participants to eliminate any learning effect. Because the simulated vehicle is equipped with a lane-keeping assistance (LKA) system in addition to the ACC system, all experiments were performed without the participants turning the steering wheel. Thus, the participants could concentrate their attention on the ACC system. In each experiment, the participant started the vehicle and turned on the ACC system. Depending on the design scenario, a specific event occurred after the host vehicle arrived at a certain location. Typical events were a sudden maneuver by an adjacent vehicle or the appearance of a road construction sign. In response to each event, the participant tried to control the vehicle by pressing the brake or gas pedal. The experimenter observed the participant’s actions and the resulting mode and state changes in the system. After each event was completed, the experimenter covered the interface on the gauge cluster and interrupted the driving simulation to ask the participant about the mode change and the reason for the answer given. The experimenter then uncovered the interface and asked the participant the same question. The experimenter noted the answers given during the experiment. The participants were expected to use the ACC system while driving. Therefore, if the active mode was canceled by pressing the brake or accelerator, the participant had to resume system operation as soon as the event was over. The participants were given no clues about the correct answers. When the driving experiments were finished, the participants were asked whether and why they felt any mode confusion. Each experimental session lasted 55 min from start to finish.

### 6.3. Apparatus

#### 6.3.1. Driving Simulator

**Figure 8:**
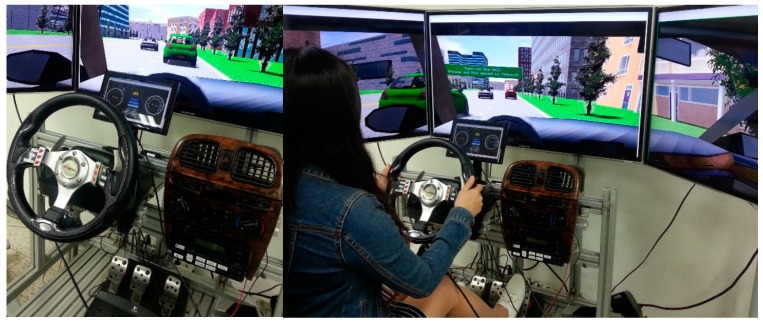
Participant operating the simulator during the experiment.

The experiments were conducted in a fixed-base driving simulator with TNO PreScan software [24], as shown in Figure 8. The simulator had three 42-inch widescreen displays that created a 130° horizontal by 25° vertical field of view and a 10-inch display for the gauge cluster. The input device was a Logitech G27 racing wheel with gas and brake pedals. The buttons on the wheel were configured to control various operations for ACC mode transitions.

**Figure 9:**
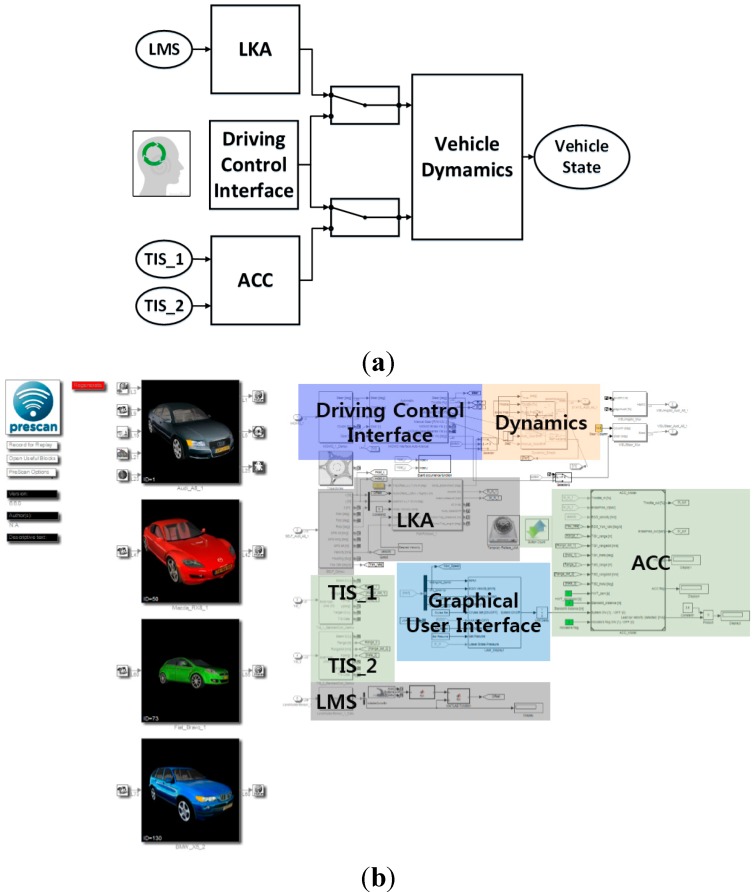
Overall system architecture of the simulated vehicle equipped with the ACC system: (**a**) system diagram; and (**b**) implementation using MATLAB and Simulink in PreScan.

In the driving simulator, the ACC system was implemented based on PreScan, using MATLAB and Simulink software by MathWorks. PreScan is a physics-based simulation platform that provides various sensor emulation functions to facilitate the rapid development of different active safety or advanced driver assistance systems. As shown in Figure 9, we designed and implemented the ACC and LKA systems and the graphical user interface using Simulink and MATLAB. Two technology-independent sensors (TISs) and one lane marker sensor (LMS), which were provided by PreScan, were used in the system. A TIS is a virtual active scan sensor that can replace a specific physical scanner, such as a radar or laser scanner. The first of the two implemented TISs had a sensor beam angle of 9° and a front detection range of 150 m. Because the beam angle was narrow and the detection range was large, this TIS was able to acquire information about faraway vehicles in the forward direction, particularly a preceding target vehicle in the same lane as the host vehicle. The second TIS had a sensor beam angle of 80° and a front detection range of 30 m. Because the beam’s angle was wide and the detection range was small, this TIS was used to detect vehicles in the forward direction in adjacent lanes or the corner section. It was also used to detect the target vehicle in the stop and go modes in a low-speed ACC system. The ACC module performed longitudinal vehicle control using the information from the two TIS systems. In addition, we designed an LKA module using Simulink and an LMS provided by PreScan. The LKA module was implemented to allow further research into user interfaces for autonomous vehicles. This module performed lateral vehicle control using lane information obtained from the LMS. The ACC module output the throttle position and brake pressure, whereas the LKA module output the steering wheel angle. These output values were delivered to the vehicle dynamics module, which calculated the dynamic and kinematic behaviors of the vehicle, such as the velocity and acceleration. Lastly, we designed the graphical user interface using MATLAB. The driving control interface module in Figure 9 is automatically activated when the Logitech G27 racing wheel is connected with the gas and brake pedals, and the Vehicle Dynamics, TIS_1, TIS_2, and LMS modules are automatically activated by linking the vehicles and sensors provided by PreScan.

#### 6.3.2. Graphical User Interface for ACC Systems

**Figure 10:**
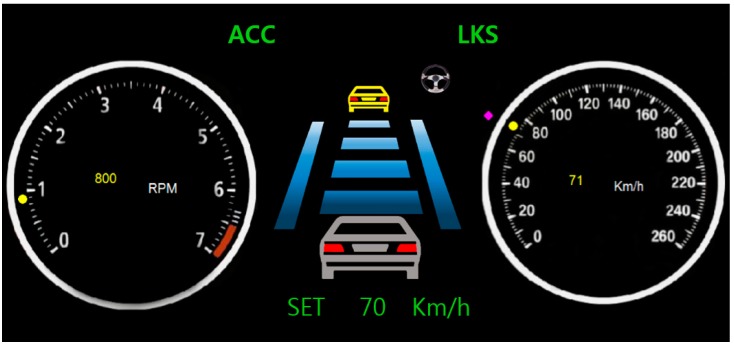
Graphical user interface for the ACC system implemented based on current interfaces.

Graphical user interfaces for the interface models were implemented in a gauge cluster and were based on the user interfaces of the ACC systems made by major automotive manufacturers, including Hyundai, Lexus, and Mercedes-Benz. In Figure 10, “ACC” and “Set 70 km/h” represent a mode in the ACC system, and the figure in the center of the gauge cluster represents the internal state of the active mode. “ACC” distinguishes the off mode from the other modes, and its colors distinguish the modes. “Set 70 km/h” distinguishes the active and canceled modes; if “Set 70 km/h” is turned off, this indicates the system is in the canceled mode, whereas if “Set 70 km/h” is turned on, this indicates it is in the active mode. Based on the traditional and new interface models shown in Figure 4b and Figure 7, respectively, we designed new user interfaces as illustrated in Table 6. The different colors for the texts “ACC” and “Set 70 km/h” are intended to allow the user to more clearly distinguish between the modes and states.

**Table 6:** Graphical user interfaces based on the traditional and new interface models for the ACC system.

State	Five-Mode Interface		Four-Mode Interface		Three-Mode Interface	
	Mode	Interface	Mode	Interface	Mode	Interface
Off	Off		Off		Off	
Armed	Armed	ACC	Armed	ACC		
Canceled	Canceled	ACC	Canceled	ACC	Canceled	
		Set 70 km/h		Set 70 km/h		ACC
Override	Override	ACC	Active			Set 70 km/h
		Set 70 km/h		ACC		
Speed Control	Active	ACC		Set 70 km/h	Active	ACC
Gap Control		Set 70 km/h				Set 70 km/h

### 6.4. Scenario

A road with six lanes (three lanes in each direction) was modeled with PreScan for use in the driving simulator experiments. The road was modeled after the Marina Bay Street Circuit of the Singapore Grand Prix, which consists of street roads in a harbor-side location. The road had straight and curved sections, and its total length was approximately 18 km. In the scenario, 10 events were designed to occur in specific regions. Table 7 shows the designed traffic situation, the expected driver operation, and the ACC mode change of each event. The modes in parentheses are those of the three-mode interface model, and the corresponding modes of the four- and five-mode models are outside the parentheses.

**Table 7:** Events and their expected mode transitions in the experimental scenario.

Event No.	Designed Traffic Situation		Expected Driver Operation	Expected Mode Transitions	
	Actor	Action		Before	After
1	V_4_	Sudden cutting in front of V_1_	Brake/None	Active	Canceled/Active
2	V_1_	Smooth braking due to traffic lights	Brake	Active	Canceled
3	V_2_	Sudden braking due to traffic lights	Brake/None	Active	Canceled/Active
4		Under construction sign	Brake	Active	Canceled
5	V_1_	Braking due to traffic lights	Brake	Active	Canceled
6	V_5_	Sudden cutting in front of V_1_ from an on-ramp	Brake/None	Active	Canceled/Active
7	V_1_	Speeding up	Pressing and releasing the gas pedal	Active	Override (Canceled)
8	V_1_	Turning off the ACC System	Pressing the ACC switch	Override (Canceled)	Off
9	V_1_	Speeding up	Pressing and releasing the gas pedal	Off	Off
10	V_1_	Turning on the ACC System	Pressing the ACC switch	Off	Armed (Active)

**Figure 11:**
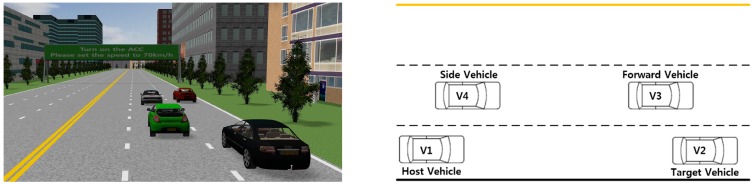
Host and surrounding vehicles in the experiments.

**Figure 12:**
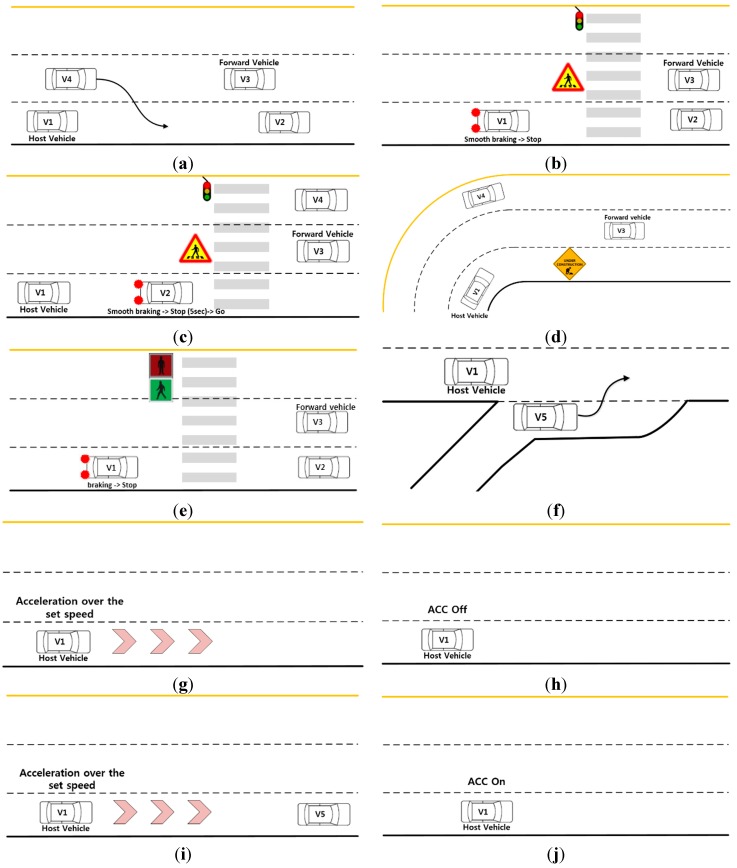
Ten events used in the driver-in-the-loop experiments to evaluate the ACC interfaces: (**a**) Event 1: V_4_ suddenly moves in front of V_1_; (**b**) Event 2: V_1_ encounters a red traffic light and must stop; (**c**) Event 3: V_2_ stops smoothly because of a red traffic light; (**d**) Event 4: V_1_ must stop because of a construction zone; (**e**) Event 5: V_1_ encounters a red traffic light and must stop suddenly; (**f**) Event 6: V_5_ moves suddenly in front of V_1_ from an entrance ramp; (**g**) Event 7: V_1_ must accelerate; (**h**) Event 8: V_1_ must turn off the ACC system; (**i**) Event 9: V_1_ must accelerate; and (**j**) Event 10: V_1_ must turn on the ACC system.

To implement the events in PreScan, three vehicles were placed around the host vehicle, V_1_, as shown in Figure 11. A target vehicle, V_2_, was in front of the host vehicle, a forward vehicle, V_3_, was in front of the host vehicle in the left lane, and a side vehicle, V_4_, was next to the host vehicle. All of the vehicles, V_2_–V_4_, moved with surrounding the host vehicle, based on the speed of the host vehicle. In addition, a merging vehicle, V_5_, moved in front of the host vehicle while entering from a ramp, as shown in Figure 12f. The surrounding vehicles were driven by a driver model called Path Follower in PreScan, and the host vehicle was driven by the participants. In each experiment, each vehicle followed a predetermined path. As the host vehicle approached a specific location, one or more of the surrounding vehicles V_2_–V_5_ exhibited predetermined behaviors, such as sudden accelerating or braking. The following ten events, illustrated in Figure 12, were included in the experimental scenarios.

## 7. Results

### 7.1. Mode Confusion Rates

The following data were collected for each event in the experiments: the participant’s operation, the actual mode after the operation, the mode that the participant predicted without looking at the ACC interface, the reason the participant thought he/she was in that mode, and the mode that the participant recognized after looking at the ACC interface.

We examined the mode confusion rates for two independent variables: the type of user interface and whether or not the participant glanced at the display. We considered three levels for the type of user interface (*i.e*., three-, four-, and five-mode models) and two levels for glancing at the display (*i.e.*, glancing and not glancing).

**Figure 13:**
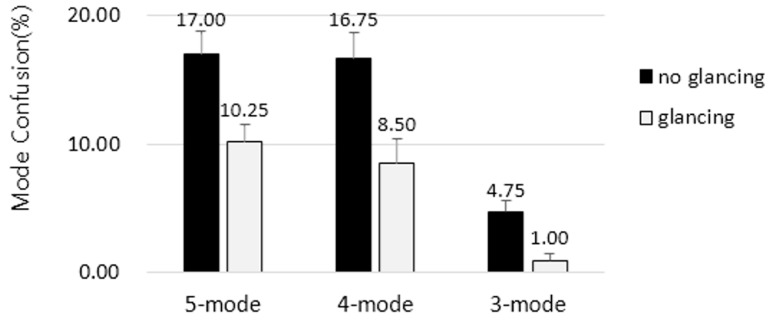
Mode confusion rates with the three investigated ACC interfaces for users glancing and not glancing at the display.

The mode confusion rates for the participants are shown in Figure 13. Repeated measures analysis of variance (ANOVA) was conducted using Minitab 16.0 to assess the significance of the two factors. Significant main effects were detected for the interface type (*F*_2,78_ = 45.20, *p* < 0.05). The differences between the three- and four-mode interfaces (*F*_1,39_ = 62.25, *p* < 0.001) and the three- and five-mode interfaces (*F*_1,39_ = 108.55, *p* < 0.001) were significant, whereas the difference between the four- and five-mode interfaces (*F*_1,39_ = 0.73, *p* = 0.394 > 0.05) was not. In addition, glancing at the display also had a significant main effect (*F*_1,39_ = 42.31, *p* < 0.05). There was no significant interaction between interface type and glancing at the display (*F*_2,78_ = 1.94, *p* = 0.146 > 0.05).

Table 8, Table 9, Table 10 and Table 11 are the confusion tables for the three ACC interfaces evaluated in this study. The confusion tables allow us to investigate how the participants recognized the changes in the mode of the ACC system during the experiments because they compare the actual modes with the modes recognized by the participants. The values in the shaded diagonal cells indicate correctly recognized states.

As Table 8 shows, in the experiments with the five-mode interface, 83.5% and 90% of the mode changes were recognized correctly without and with glancing at the display, respectively. As Table 9 shows, for the four-mode interface, 83.5% and 91.8% of the mode changes were recognized correctly without and with glancing, respectively. However, as Table 10 shows, for the three-mode interface, 95.5% and 99% of the mode changes were recognized correctly without and with glancing, respectively. This is an amazing result in the design of the ACC interface. This outstanding result is attributable to the conciseness and clarity of a user interface with just three simple modes: off, canceled, and active.

**Table 8:** Mode confusion rates before and after glancing at the five-mode interface in the experiments.

		Modes Recognized by Drivers												
Actual Modes	Mode	Off		Armed		Canceled		Override		Active		Mode Confusion		Total
		Before	After	Before	After	Before	After	Before	After	Before	After	Before	after	
	Off	83 (100%)	84 (100%)	0 (0%)	0 (0%)	0 (0%)	0 (0%)	0 (0%)	0 (0%)	0 (0%)	0 (0%)	0 (0%)	0 (0%)	167
	Armed	0 (0%)	0 (0%)	20 (47%)	25 (58%)	5 (11%)	11 (26%)	3 (7%)	3 (7%)	15 (35%)	4 (9%)	23 (53%)	18 (42%)	86
	Canceled	0 (0%)	1 (1%)	19 (13%)	9 (6%)	125 (84%)	137 (93%)	1 (1%)	0 (0%)	3 (2%)	0 (0%)	23 (16%)	10 (7%)	295
	Override	0 (0%)	0 (0%)	6 (17%)	6 (17%)	1 (3%)	2 (6%)	26 (74%)	27 (77%)	2 (6%)	0 (0%)	9 (26%)	8 (23%)	70
	Active	0 (0%)	0 (0%)	2 (2%)	0 (0%)	6 (7%)	3 (3%)	3 (3%)	1 (1%)	80 (88%)	87 (96%)	11 (12%)	4 (4%)	182
	Total	83	85	47	40	137	153	33	31	100	91	66	40	800

**Table 9:** Mode confusion rates before and after glancing at the four-mode interface in the experiments.

		Modes Recognized by Drivers													
Actual Modes	Mode	Off			Armed				Canceled		Active		Mode Confusion		Total
		Before		After	Before		After		Before	After	Before	After	Before	after	
	Off	79 (99%)		80 (100%)	0 (0%)		0 (0%)		0 (0%)	0 (0%)	1 (1%)	0 (0%)	1 (1%)	0 (0%)	160
	Armed		0 (0%)	0 (0%)		29 (66%)		33 (75%)	6 (14%)	10 (23%)	9 (20%)	1 (2%)	15 (34%)	11 (25%)	88
	Canceled		1 (1%)	0 (0%)		20 (15%)		14 (11%)	103 (82%)	114 (89%)	2 (2%)	0 (0%)	23 (18%)	14 (11%)	254
	Active		0 (0%)	0 (0%)		13 (9%)		4 (3%)	14 (9%)	4 (3%)	123 (82%)	140 (94%)	27 (18%)	8 (6%)	298
	Total		80	80		62		51	123	128	135	141	66	33	800

**Table 10:** Mode confusion rates before and after glancing at the three-mode interface in the experiments.

		Modes Recognized by Drivers								
Actual Modes	Mode	Off		Canceled		Active		Mode Confusion		Total
		Before	After	Before	After	Before	After	Before	after	
	Off	81 (99%)	82 (100%)	1 (1%)	0 (0%)	0 (0%)	0 (0%)	1 (1%)	0 (0%)	164
	Canceled	0 (0%)	0 (0%)	155 (92%)	164 (98%)	13 (8%)	4 (2%)	13 (8%)	4 (2%)	336
	Active	0 (0%)	0 (0%)	4 (3%)	0 (0%)	146 (97%)	150 (100%)	4 (3%)	0 (0%)	300
	Total	81	82	160	164	159	154	18	4	800

**Table 11:** Questions and responses after experiments with the three-, four-, and five-mode ACC interfaces.

Question	Response	No. of Participants		
		Five Mode	Four Mode	Three Mode
Did you have mode confusion during the experiment?	Yes	31	19	2
Which modes made you confused?	Armed-Canceled	18	11	NA
	Armed-Active	6	5	NA
	Armed-Override	5	NA	NA
	Armed-Canceled-Override	2	NA	NA
	Canceled-Active	2	7	2
	Canceled-Override-Off	5	NA	NA
	Canceled-Override	2	NA	NA
Why were you confused?	Confused in the mode definitions	25	11	2
	Confused in the button meanings	5	5	NA
	Had no idea about how to do after pushing the pedals	12	8	NA
What did you do when you had the mode confusion?	Looking at the interface display	9	7	1
	Turning off and on the ACC	12	5	1

With the four- and five-mode interfaces, the highest mode confusion rate occurred in the armed mode, regardless of whether the display was viewed. Some participants believed that the state changed immediately into the active mode and that the speed was set when they pressed the ACC button. Other participants confused armed with canceled because they were not actually aware of the difference between the two states. However, we expected that looking at the display of the ACC interface might reduce the incidence of mode confusion. The experimental results show that the cases in which the armed mode was confused with the active mode decreased from 35% to 9% and from 20% to 2% in the five- and four-mode interfaces, respectively, after the participants looked at the display. However, the cases in which the armed mode was confused with the canceled mode increased from 11% to 26% and from 14% to 23% in the five- and four-mode interfaces, respectively, after the participants looked at the display. This confusion is believed to occur because of the similarity of the orange and red text colors that indicate the armed and canceled modes, respectively, and the fact that the participants did not understand that “Set __ km/h” was related to the operation mode. The set speed was not present in the armed mode, but it was present in the canceled mode. In addition, the confused participants did not sufficiently understand the difference between the armed and canceled modes. Therefore, if possible, the armed and canceled modes should be merged to prevent mode confusion. The experimental results for the three-mode interface support this claim.

The confusion rates in the canceled mode were 16%, 18%, and 8% with the five-, four-, and three-mode interfaces, respectively, without looking at the display. The participants who confused canceled with armed misunderstood that the ACC’s set speed would be reset when they pressed the brake. A single participant confused canceled with off and answered that pressing the brake pedal turned off the ACC. The 18 participants who confused canceled with active answered that they could not know whether the ACC was active or not without looking at the interface. After looking at the interface display, mode confusion was completely removed in the active mode in the five- and four-mode interfaces. However, in the three-mode interface, mode confusion still existed. Four confused participants answered that the active mode included the canceled mode.

The confusion rates of the override mode were 26% and 23% in the five-mode interface before and after looking at the interface display, respectively. Mode confusion was not reduced because the participants who answered incorrectly did not properly understand the definitions of the modes.

The confusion rates of the active mode were 12%, 18%, and 3% for the five-, four-, and three-mode interfaces, respectively, without looking at the interface display. Instances of mode confusion occurred mostly after Event 6, in which the mode changed from canceled to active when the participant pressed the resume button. The active mode was confused with various modes. Some participants who confused active with armed did not press the resume button but rather the set button. Other participants who confused active with canceled did not remember that they had to press the resume button after pressing the brake. Other participants who confused active with override thought that the override mode simply accelerated the vehicle regardless of whether the gas pedal was pressed or not. One of the reasons for this misunderstanding was the existence of too many modes in the five-mode interface. After looking at the interface display, mode confusion was completely or almost completely removed in the active mode.

**Figure 14:**
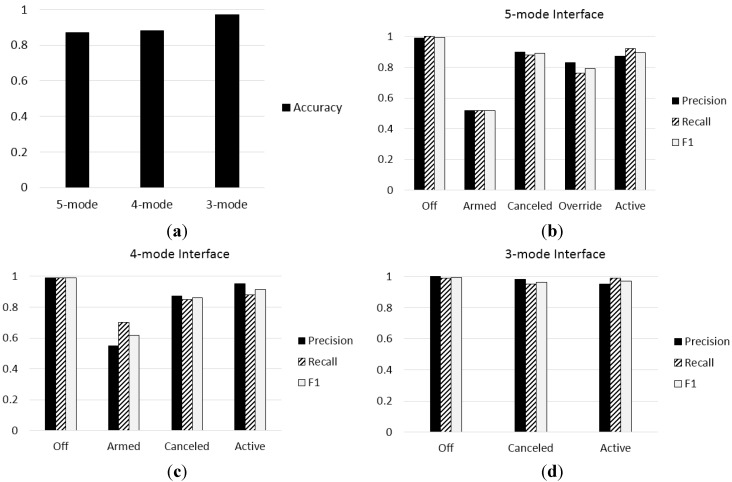
Performance evaluation results for the three types of interfaces: (**a**) accuracy; (**b**) precision, recall, and F1 measure of the five-mode interface; (**c**) precision, recall, and F1 measure of the four-mode interface; and (**d**) precision, recall, and F1 measure of the three-mode interface.

Typical performance evaluation metrics, such as accuracy, precision, recall, and the F1 measure [25], were evaluated and are summarized in Figure 14. The accuracies of the five- and four-mode interfaces are 0.87 and 0.88, respectively, whereas the accuracy of the three-mode interface is 0.97. As shown in Figure 14, the three-mode interface shows significantly better performance than the other interfaces. In the three-mode interface, incorrect mode recognition decreased dramatically because three different modes that indicate the standby status of the ACC system (*i.e*., armed, canceled, and override modes) were merged into one mode (*i.e*., canceled mode).

### 7.2. Questionnaire Survey

#### 7.2.1. Summary of Questionnaire Results after Each Experiment

After performing the experiment with each interface, the participants completed a questionnaire of four questions concerning whether the participant was confused during the experiment, which modes confused him/her, why he/she was confused, and which action he/she took when he/she was confused. Their responses for the three-, four-, and five-mode interfaces are summarized in Table 11.

As Table 11 shows, for the five-mode ACC interface, 31 participants experienced instances of mode confusion. These instances were classified into seven types: armed-canceled, armed-active, armed-override, armed-canceled-override, canceled-override, canceled-override-off, and canceled-active. For the four-mode interface, 19 participants experienced confusion during mode changes, and the instances of mode confusion were classified into three types: armed-canceled, armed-active, and canceled-active. For the three-mode interface, two participants were confused between the canceled and active modes (canceled-active confusion). Therefore, a total of seven different types of mode confusion occurred during the experiments. Among these types, the mode confusion between the armed and canceled modes was the most common, occurring for 18 and 11 participants in the five- and four-mode interfaces, respectively. The participants answered that this mode confusion occurred because each mode definition or button meaning was confused or they did not know what to do in the armed or canceled mode. The confusion of mode definition was the most common reason for mode confusion, as reported in the questionnaire by 25, 11, and two participants for the five-, four-, and three-mode interfaces. In the questionnaire, 17 participants answered that after experiencing mode confusion, they could correctly recognize the modes by viewing the interface display and take appropriate action. However, 12, five and one participants in the five-, four- and three-modes who incorrectly recognized a mode even after viewing the interface display solved their mode confusion by turning the ACC system off and on again. The rest of the participants who experienced mode confusion asked for help from the experiment coordinator. To summarize, the existence of both the armed and canceled modes can cause drivers to experience mode confusion.

#### 7.2.2. Summary of Questionnaire Results after All Experiments

After all of the experiments were completed, the participants were asked to complete a questionnaire concerning the following points: which interface the participant thought was the best among the three ACC interfaces, why he/she selected this interface, which interface he/she thought was the worst among the three interfaces, and why he/she selected this interface. The participants’ responses are summarized in Table 12.

**Table 12:** Questions and responses after all experiments were completed.

Question	Response	No. of Participants
Which one is the best among the three user interfaces?	Three-mode interface	40
Why did you make your selection for the best interface?	Easy to use	10
	Comfortable	5
	Reducing driving workload	25
Which one is the worst among the three interfaces? (Which one caused the most mode confusion to you?)	Four-mode interface	5
	Five-mode interface	35
Why did you select it as the worst interface?	Armed mode is not necessary	38
	Override mode is not necessary	4
	Too excessive number of modes	19
	Increasing driving workload	14

As shown in Table 12, the participants unanimously selected the three-mode ACC interface as the best. They felt it was easy to observe the current mode and found the use of this interface mentally comfortable because it has few operational modes and they did not need to remember their past actions. In addition, some answered that the use of this interface reduced their driving workload because the information displayed on the three-mode interface was delivered accurately without any confusion when they controlled the ACC system. Among the 40 participants, 35 chose the five-mode interface as the worst interface, and the remaining five chose the four-mode interface. The participants who chose the four-mode interface as the worst were of the opinion that the armed mode was not necessary and did not understand the difference between the armed and canceled modes during the experiments. The participants who selected the five-mode interface as the worst remarked that the modes were subdivided excessively, which caused mode confusion. Some participants felt their driving workload was greater when they used the five-mode ACC interface than when they did not because they had to recall their past actions. Thus, they thought that the armed, canceled, and override modes were not necessary. These results show that the participants want the clearest and most concise user interface for the ACC system.

## 8. Discussion

The experimental results showed that the three-mode interface was the most effective of the three interfaces in reducing mode confusion. The total rates of mode confusion with the five- and four-mode interfaces were 13% and 12%, respectively, whereas that with the three-mode interface was only 3%. As described in Section 5, we proposed a new user interface design methodology for automated systems and applied it to ACC interfaces, and this methodology consists of three main steps: designing the machine and interface models, testing the compatibility between the two models, and, if they are incompatible, redesigning one or both of the models. Although two correct interfaces were produced on the basis of this methodology, their performances were significantly different. Now, we would like to determine why the five-mode interface did not show any improvement over the traditional four-mode interface in spite of its correctness and why the three-mode interface showed such significant improvement.

First, contrary to expectations, the experimental results showed that the difference in the performances of the four- and five-mode interfaces was not significant. As the four-mode interface had incompatible state transitions in the active mode, we expected that its mode confusion rates would be higher than those of the five-mode interface. However, their difference was not statistically significant. In the active mode of the four-mode interface, the override state was incompatible with the speed control and gap control states. The confusion rate between the active and canceled modes was 6.0% in the four-mode interface. However, in the five-mode interface, the confusion rate between the override/active and canceled modes was 4.8%. Because the mode confusion rate due to the incompatibility of the active mode was low, the effect of the removal of the incompatible states should have been low as well.

Conversely, the experimental results show that the difference between the mode confusion rates of the three- and four-mode interfaces was significant, as expected. In the four- and five-mode interfaces, regardless of whether the user glanced at the display, the highest mode confusion rate was observed in the armed mode. Conversely, the three-mode interface showed very low confusion rates in the canceled mode. The three-mode interface was designed by following the interface-centric approach where the machine model was modified according to the interface model if the two were incompatible. This approach guarantees a user-friendly interface that can provide users a more compact, clear, convenient, and safe ACC interface.

## 9. Conclusions

In this study, we proposed a new interface design methodology and applied it to the design of driver interfaces for an ACC system. In this methodology, the compatibility between the initial machine and interface models is reviewed using the proposed criteria, and if the two are incompatible, one or both of the models is/are modified to make them compatible. Two different methods of modification were used: modifying the machine model and modifying the interface model. The results of the driver-in-the-loop experiments support the hypothesis that modifying the machine model to resolve the incompatibility would produce a more compact and easy-to-understand user interface than modifying the interface model.

For future work, autonomous vehicle issues that have recently emerged need to be addressed. Because of the recent development of autonomous vehicles, such as Google cars, there is an urgent need to develop optimal user interfaces for multiple levels of automation in autonomous vehicles. The United States National Highway Traffic Safety Administration (US NHTSA) has defined five levels of vehicle automation. As the level of automation increases, the role of the driver shifts from primary driving controller to supervisory controller. Future vehicles will automatically set the automation level for a driver or a system depending on the state of the driver, vehicle, and environment. Thus, drivers should not be surprised or confused by the automation levels or system modes. Based on the current NHTSA criteria, the present study only addressed low levels of automation. Therefore, it is necessary to extend this study to higher levels of autonomy in autonomous vehicles [26,27], In addition, recently developed multi-modal interaction technologies must be applied to the driver-vehicle interface to reduce not only driver distraction but also mode confusion and automation surprise [28,29,30,31,32].
